# A Novel Approach to Measuring Muscle Mechanics in Vehicle Collision Conditions

**DOI:** 10.3390/s17061389

**Published:** 2017-06-14

**Authors:** Simon Krašna, Srđan Đorđević, Marija Hribernik, Ana Trajkovski

**Affiliations:** 1 Faculty of Mechanical Engineering, University of Ljubljana, Aškerčeva cesta 6, 1000 Ljubljana, Slovenia; ana.trajkovski@fs.uni-lj.si; 2 TMG-BMC d.o.o., Štihova ulica 24, 1000 Ljubljana, Slovenia; srdjand@tmg.si; 3 Faculty of Medicine, University of Ljubljana, Korytkova ulica 2, 1000 Ljubljana, Slovenia; marjana.hribernik@mf.uni-lj.si

**Keywords:** biomechanics, vehicle occupant, impact, in vivo, active muscle

## Abstract

The aim of the study was to evaluate a novel approach to measuring neck muscle load and activity in vehicle collision conditions. A series of sled tests were performed on 10 healthy volunteers at three severity levels to simulate low-severity frontal impacts. Electrical activity—electromyography (EMG)—and muscle mechanical tension was measured bilaterally on the upper trapezius. A novel mechanical contraction (MC) sensor was used to measure the tension on the muscle surface. The neck extensor loads were estimated based on the inverse dynamics approach. The results showed strong linear correlation (Pearson’s coefficient ${\bar{r}}_{P}$ = 0.821) between the estimated neck muscle load and the muscle tension measured with the MC sensor. The peak of the estimated neck muscle force delayed 0.2 ± 30.6 ms on average vs. the peak MC sensor signal compared to the average delay of 61.8 ± 37.4 ms vs. the peak EMG signal. The observed differences in EMG and MC sensor collected signals indicate that the MC sensor offers an additional insight into the analysis of the neck muscle load and activity in impact conditions. This approach enables a more detailed assessment of the muscle-tendon complex load of a vehicle occupant in pre-impact and impact conditions.

## 1. Introduction

Development and application of vehicle active safety systems received a great deal of attention from researchers and the automotive industry. Muscle activity can affect the body posture and dynamic response of the occupant’s body in low-velocity vehicle collisions, evasive braking, and steering manoeuvres (as well as deployment of autonomous collision avoidance systems) [1,2,3,4,5,6]. Bracing and occupant body dynamics may also influence the injury outcome in low-severity impacts [7]. Moreover, real-life occupant response can differ from the measured response of anthropometric test devices and postmortem human subjects that are commonly used for validation in human body modelling. Comparison of volunteer and simulated human body model response prior to a vehicle collision showed that both the muscle activation level and timing could have a significant effect on the head-neck dynamics, particularly in frontal impacts [8,9,10]. Based on simulating a validated human body model, dynamic conditions and critical situations can be efficiently analysed without posing a risk to the volunteers. Estimating skeletal muscle forces that influence human body motion can provide an insight into the average or person-specific active response, injury probability, and interaction of the human body with safety restraints, as well as assessment of driving ergonomics and comfort [11,12,13].

Direct measuring of muscle forces is mostly inappropriate due to invasiveness. Instead, muscle forces can be estimated via computational modelling and analysis of human body dynamics. Typically, the Hill-type muscle model [14] with muscle activation control [15] is implemented for simulating muscular forces that generate joint moments to control human body posture and motion. Several assumptions and input parameters that are difficult to determine accurately are needed for the muscle model, such as muscle physiology, interaction with the surrounding anatomical structures during motion, muscle activation, and load distribution over redundant muscles. Based on the measured body segment kinematics and external loads data, inverse dynamic analysis can retrieve the joint moment generated by a group of muscles; the individual muscle forces can then be estimated with the help of optimisation methods to resolve the force distribution between the redundant muscles acting on the joint [15,16,17,18,19,20]. In forward dynamic analysis of the musculoskeletal model, muscle activations can be optimised to produce muscle forces that minimize the difference between the simulated and the measured human body motion.

Another widely used method for estimating muscle force and activity is electromyography (EMG), where muscle electrical activity is measured through surface or intra-muscular wire electrodes. The recorded and post-processed EMG signals represent input parameters for muscle activation and contraction dynamics generating the muscle force [14,21,22]. The EMG data may be applied together with dynamic analysis and optimisation of the musculoskeletal model [22,23,24,25]. Surface EMG is preferably used for larger superficial muscles, while measuring deep muscles with fine-wire electrodes is more difficult due to invasiveness and the possibility that the signal recorded represents only local muscle activity and is affected by movement artefacts in dynamic conditions [26,27].

Measuring the relevant EMG signal and troublesome interpretation of recorded EMG signals may present a challenge. De Luca [28,29] and Hedenstierna et al. [26] listed numerous factors, tightly inter-related, that influence the usability of EMG recordings. For impact conditions, particularly the timing and amplitude of the EMG signal may be considered. The delay between the EMG signal and the force build-up depends on the fibre-type composition, the muscle unit firing rate and visco-elastic properties of the muscle-tendon complex. The EMG signal/force relationship is generally nonlinear and cannot be estimated directly for several reasons including muscle physiology, imperfect detection of electrical activity, and muscle force calculation. Based on adjusting the muscle activity of a bracing occupant, Bose and Crandall [30] showed that including realistic muscle activity in the restraint design and analysis is of vital importance. Muscle force in isometric conditions can be estimated using EMG with high accuracy, while muscle length and contraction velocity affect the muscle force generation in dynamic conditions, which may compromise the ability of EMG to predict the muscle force [31]. Additionally, the action of the whole muscle-tendon complex is preferred in analysis of dynamic events where isolated muscle activity measured by EMG gives only limited information [32]. In either case, the human body active response must be validated and calibrated to experimental data from volunteer body dynamics and measured muscle activity. The usual approach is to minimize the difference between the simulated and postmortem human subject or volunteer response. Siegmund et al. [33] suggested that for the posterior neck muscles in vivo data should be used for neck model validation instead of minimising the objective cost function. Anderst et al. [34] used the inverse dynamics approach to estimate neck muscle forces, where in vivo motion data was used to drive a numerical model of the head-neck complex, but for slow voluntary motion only. Funk et al. [35] verified that the inverse dynamics approach can be used for the neck load estimation in case of impact forces acting to the head, while Seacrist et al. [36] and Beeman et al. [2,3,4] analysed volunteers’ neck loads during low-velocity frontal impacts. However, the joint moment results from the action of the whole muscle group, the electrical activity of which cannot be assessed with a single EMG electrode, making it difficult to correlate the EMG signal to the body dynamic response directly. Ólafsdóttir et al. [37] obtained experimental data using surface and needle EMG electrodes on the cervical muscles, and found a large range of neck muscle activity levels during perturbations with low pulse in different directions.

The method of mechanomyography uses the mechanical contraction (MC) sensor attached to the surface of the skin over the muscle [38]. The indenting tip of the MC sensor protrudes into the surface, effectively measuring the muscle tension in a perpendicular direction and consequently longitudinal load. Comparison of the MC sensor indentation force to EMG and the muscle force generated showed highly linear correlation for isometric contractions [39], with the MC sensor having advantages regarding the ease of use and taking into account muscle deformations. The MC sensor potentially offers better understanding of the muscle load and activity in human body motion. In addition to EMG, the MC sensor method enables estimation of the whole muscle-tendon response consisting of active and passive components. The aim of the presented study was to evaluate the application of the MC sensor in impact biomechanics—in particular to evaluate and validate the human muscle loading—and to identify output parameters of the MC sensors, complementary to EMG, accelerometers, and motion capture.

## 2. Materials and Methods

### 2.1. Experimental Setup and Data Processing

**Figure 1:**
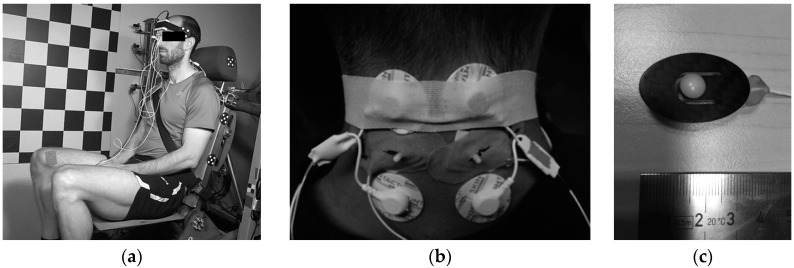
(**a**) A volunteer seated on the sled test device; (**b**) Two MC sensors and EMG surface electrode pairs attached bilaterally to the upper trapezius; (**c**) MC sensor-bottom side with indenting tip (5 mm radius).

Ten volunteers were involved in this study: seven males and three females (age 28.2 ± 6.2). The volunteers’ dynamic response was tested in low-velocity frontal impacts. The study was approved by the national Committee of Ethics, and informed consent was declared from the volunteers. The impact tests were performed on a sled test device with 10° inclined direction of travel similar to the apparatus used by Ejima et al. [40]. The deceleration pulse was generated by the sled impact to the barrier and measured with one-axis accelerometer attached to the sled. The volunteers were seated on a rigid seat (Figure 1a) with the seat back tilted at 15°, the seat base levelled horizontally, and the foot rest at 45°. A standard 3-point safety belt was used, with a load cell measuring the tension force in the upper belt segment (FGB Instrumentation, FN 4060 TI XAM-MV, Les Clayes-sous-Bois, France).

The volunteers were equipped with a 3-axis accelerometer on the forehead and an MXT marker attached to the side of the head, considered as a rigid body. A high-speed Photron FastCam Ultima 512 camera (1000 fps) was attached to the sled perpendicular to the sagittal plane. Two MC sensors (MC-System, TMG-BMC, Ljubljana, Slovenia) were attached posteriorly to the upper trapezius muscle to the left and right hand side (Figure 1b), together with two surface EMG electrodes (Skintact F-301, Innsbruck, Austria). As the MC sensor's indenting tip (Figure 1c) protruded the skin surface, the piezoresistive silicon force sensor with known sensitivity [38] returned the output signal proportional to the indenting force. To ensure non-zero indentation force during the test, preload ~1 N was applied to the tip with adjusting screw. EMG data were collected with Biosignalsplux EMG (PLUX, Lisbon, Portugal). The EMG signal was sampled at a 1 kHz sampling rate and band-pass filtered at 10–400 Hz. To estimate the linear envelope of the EMG signal, we calculated the root mean square value (full-wave, 25 ms window) and smoothed the signal using a 15 Hz 6th-order low-pass zero-phase Butterworth filter. The MC signal was sampled at 1 kHz and low-pass filtered (20 Hz, 6th-order zero-phase Butterworth filter). The measured signals were saved within a portable unit containing data logger. The trigger was set to the impact of the sled and the barrier, defining time 0. The initial values of the MC sensor and the EMG signals were set to 0 based on averaged recorded signals 3 s prior to the impact. Data was analysed for the interval from 0 to 0.9 s after the impact. Additionally, two MC sensors were attached to the vastus medialis muscle. For the current paper, only the subset of the measured data that is relevant to the focus of the study is presented in the following sections.

The volunteers were instructed to hold a comfortable position on the seat and to relax during the tests. Three low-velocity impact scenarios were tested: low (1.7 g), medium (2.6 g), and high (3.8 g) impact pulse. The sled impact pulse was within the range of other studies on the volunteer dynamic response in frontal impacts, such as those by Beeman et al. (2.5–4.7g) [2] and Seacrist et al. (3.8 g) [36], who used inverse dynamics analysis of the neck loads, as well as other experimental and numerical studies on the neck muscle activity [5,8,37,41,42]. Table 1 shows the average parameters (mean ± standard deviation (SD)) of the impact pulse used. 

**Figure 2:**
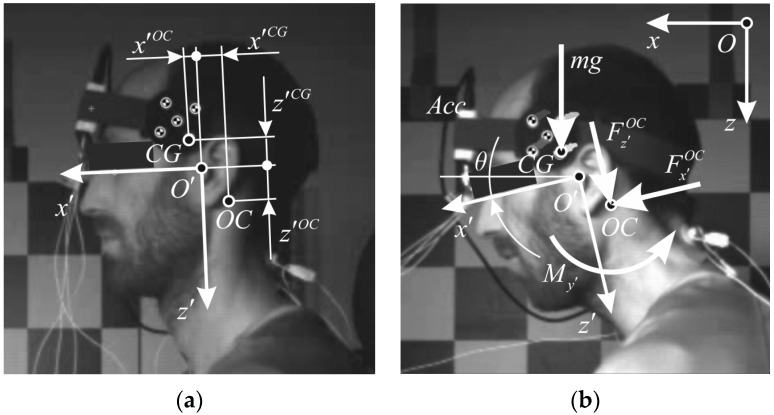
(**a**) Local coordinate system of the head: origin O′, center of gravity (*CG*), occipital condyle (*OC*); (**b**) external loads on the head-neck joint at *OC*.

Based on tracking the MXT marker, planar motion analysis of the head was performed using an open source software tool Kinovea (v. 0.8.15, www.kinovea.org). A local coordinate system with the origin O′ was defined in the mid-sagittal plane, according to the SAE J211 convention, with the local x′-axis on the Frankurt plane, directed anteriorly from the external auditory meatus, and the local z′-axis directed perpendicularly downward (Figure 2). Direction of the global x-axis was set horizontally forward, whereas global z-axis was pointed vertically with the line of gravity g. For the motion analysis, proprietary modules were programmed in MATLAB (Mathworks, Natick, MA, USA). Smoothing spline was used to the time history data of the tracked marker. The smoothing parameter was set to *p* = 0.995, which reduced the noise and preserved the shape of the tracked data. The head kinematics was estimated using least-square minimization and singular value decomposition [43]. Global position and orientation, x, z, θ, were differentiated to obtain translational and angular velocity ($v_{x},\;v_{z},\;\omega$) and acceleration $(a_{x},\;a_{z},\;\alpha$) of the head. The head rotation angle θ was defined as positive during the neck flexion.

### 2.2. Calculations

Data on the head mass and geometrical properties that were used in our study were collected and reviewed by Yoganandan et al. [44] and also include the results of Beier et al. [45] and Plaga et al. [46] (Table 2).

The loads between the head and the neck at occipital condyles (*OC*) were estimated based on the inverse dynamics approach. Since the head is considered as a rigid body, the centre of gravity (*CG*) acceleration is,

(1) $${\vec{a}}^{CG}=\vec{a}+\vec{\alpha }\times {\vec{s}}^{CG}+\vec{\omega }\times \left(\vec{\omega }\times {\vec{s}}^{CG}\right),$$

which in scalar form leads to,

(2) $$a_{x}^{CG}=a_{x}+\alpha z^{CG}-\omega^{2}x^{CG},$$

(3) $$a_{z}^{CG}=a_{z}+\alpha x^{CG}-\omega^{2}z^{CG},$$

Rotation matrix is used to transform accelerations, Equations (2) and (3), from the global coordinate system to the local one, defined anatomically (Figure 2),

(4) $$\left[\begin{array}{c} a_{x\prime }^{CG}\\ a_{z\prime }^{CG} \end{array}\right]=\left[\begin{array}{cc} \cos\;\theta & \sin\;\theta \\ -\sin\;\theta & \cos\;\theta \end{array}\right]\left[\begin{array}{c} a_{x}^{CG}\\ a_{z}^{CG} \end{array}\right]$$

According to Newton’s equations of dynamic equilibrium, loads acting on the head at *OC* are,

(5) $$F_{x\prime }^{OC}=ma_{x\prime }^{CG}-mg\sin\theta ,$$

(6) $$F_{z\prime }^{OC}=ma_{z\prime }^{CG}-mg\cos\theta ,$$

(7) $$M_{y\prime }=I_{y\prime }^{O\prime }\alpha -F_{x^{\prime }}^{OC}z^{\prime OC}-F_{z^{\prime }}^{OC}x^{\prime OC}-\;mg\sin\;\theta z^{\prime CG}-mg\cos\;\theta x^{\prime CG}.$$

where shear force $F_{x\prime }^{OC}$, axial force $F_{z\prime }^{OC}$, and bending moment $M_{y\prime }$ are expressed in the local coordinate system of the head, and the moment of inertia is corrected for the distance between CG and O′, from $I_{y\prime }^{CG}$ (Table 2) to $I_{y\prime }^{O\prime }$.

The occipital condyle (*OC*) is considered a simple rotational joint, where equilibrium force and torque can be calculated based on the head kinematics and estimated mass properties. This simple model neglects the complexity of the head-neck region and the load distribution over other neck structures. Yet, it is used in volunteer testing, where the neck muscle force cannot be measured directly, but it is correlated to the *OC* load. For the study, it was assumed that the bending moment $M_{y}$ around *OC* exerted by the external loading is predominantly counteracted by the neck extensor muscles attached to the skull. Anderst et al. [34] estimated the moment arms of the neck extensors for trapezius being 4.8 cm in the neutral posture and decreasing almost linearly with the flexion angle θ with coefficient −0.0325, which was chosen for our analysis to estimate the equilibrium muscle force.

### 2.3. Statistical Methods

The relation of the volunteers’ dynamic response to the measured activity of the neck musculature was statistically analysed. The level of statistical significance was specified at *α* = 0.05. For the head kinematic parameters and *OC* loads, the peak values and the time of the peak values occurrence were analysed. The Spearman’s correlation coefficient was used to examine correlations between the peak values and the timing of head kinematic parameters, *OC* loads and signals from the EMG and MC sensor, with the total sample size of *N* = 60 tests performed by the volunteers at three levels of impact. The correlation between EMG signals and the MC sensor indentation force on one side and the estimated neck muscle load on the other side was evaluated with the Pearson’s correlation coefficient. The Pearson’s correlation coefficient of the neck muscle force to the MC sensor indentation force was computed for each test conducted in our study, where 10 subjects performed two sequential tests, resulting in 20 tests performed in low-, medium-, and high-severity impact conditions.

## 3. Results

The analysis of the sled test data showed that the dynamic response of the volunteers was repeatable and consistent with the results of other studies. Due to the body size variations in volunteers, the initial position of the head varied considerably among the volunteers. However, the initial head position shifted progressively with increased impact severity (Table 3). With respect to the low-severity conditions, the head moved posteriorly for medium (*p* = 0.004) and high (*p* < 0.001) severity. Also, the Frankfurt plane angle rotated backwards significantly for medium- (*p* = 0.001) and high- (*p* = 0.005) severity conditions.

Time of the peak magnitude decreased with impact severity for both the EMG and the MC sensor signals, indicating a shorter reflex response. The two-sample *t*-test showed no significant difference between the left and the right hand side for the EMG (peak: *p* > 0.1996, timing: *p* > 0.5983) and the MC signals (peak: *p* > 0.2905, timing: *p* > 0.2130), so data from both sides were averaged.

**Figure 3:**
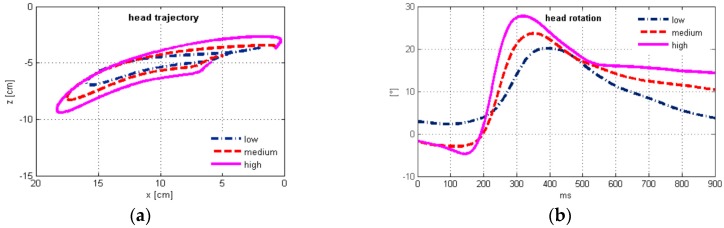

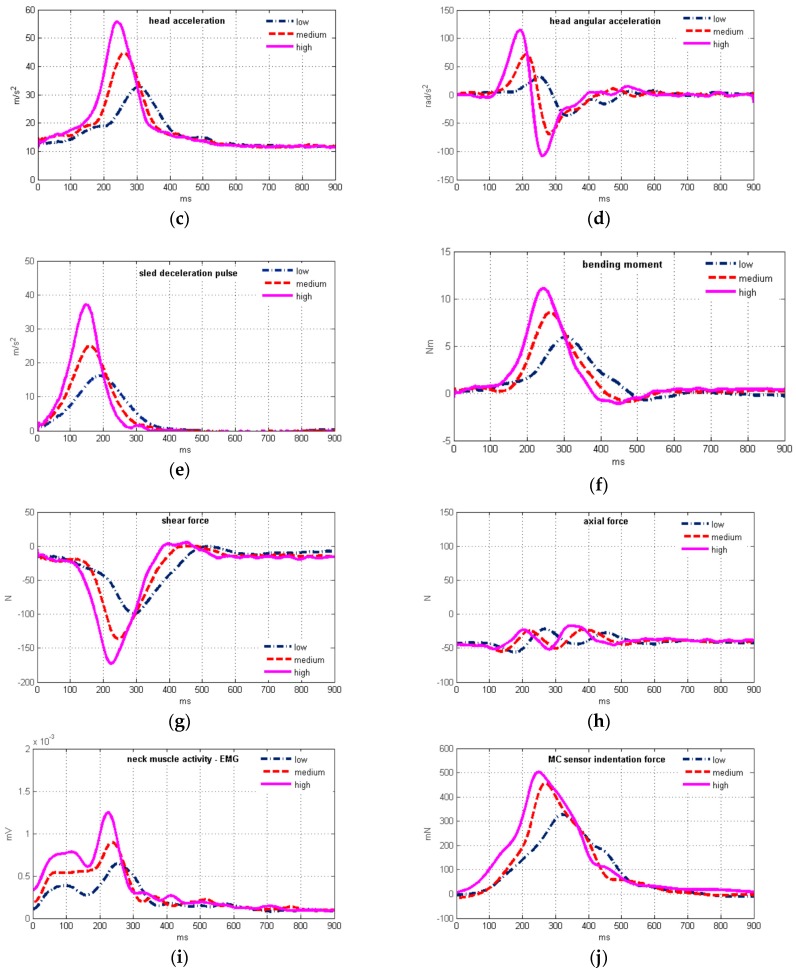
Average time history of (**a**) head displacements; (**b**) head rotation; (**c**) head translational acceleration; (**d**) head angular acceleration; (**e**) sled deceleration pulse; (**f**) *OC* bending moment; (**g**) *OC* shear force; (**h**) *OC* axial force; (**i**) EMG signals of upper trapezius activity; (**j**) MC sensor. The plots are presented for low (1.7 g), medium (2.6 g), and high impact severity level (3.8 g).

The EMG mean signal exhibited two peaks (Figure 3i). The first peak appeared at ~100 ms for all impact conditions, when small negative rotation occurred indicating neck extension. A detailed review of the individual tests revealed that this response in the early phase of the impact is not entirely consistent for all the volunteers included. The mean difference between peak timing of the MC and the EMG signals decreased with the impact severity, from 71.9 ± 37.4 ms (low) to 62.0 ± 36.7 ms (medium) and 50.6 ± 35.5 ms (high), which was expected, since with increased severity the dynamic response of the occupant’s body is faster, while having less time for an active response characterized by muscle electrical activity.

Plots of the average time history for the head motion, sled pulse, neck loads, and the EMG and MC sensor signals are depicted in Figure 3. Mean values of the peak magnitudes and their timing were determined for the main parameters of the volunteers’ dynamic response, together with their Spearman correlation between the EMG and MC sensor output (Table 4).

The peak value timing of the neck axial force was not significantly correlated to the timing of the peak EMG and MC signals. The shear force, the bending moment, and the head acceleration showed stronger correlation regarding their peak values and timing. The peak head excursion was moderately correlated to the peak MC signal, while no significant correlation was found between the peak values of the head rotation and EMG signal. The peak value and timing of the neck muscle force showed more correlation to the MC than to the EMG signal.

The average delay (*N* = 60) of the neck muscle force was 61.8 ± 37.4 ms vs. the EMG signal, while the delay vs. the MC sensor was close to zero, 0.2 ± 30.6 ms (Table 5). The peak bending moment slightly preceded the peak MC signal, −3.4 ± 29.1 ms, and delayed 58.0 ± 35.2 ms vs. the EMG signal. The estimated shear force preceded the peak MC signal, −23.1 ± 24.0 ms. Additionally, the *t*-test showed (Table 6) that the peak timing of the head motion parameters, the OC loads, and the neck muscle force were significantly different from the peak timing of the EMG signal. However, in none of the impact conditions was the MC sensor signal significantly different from the neck muscle force and the bending moment.

Generally, the peak values of head motion parameters and *OC* loads exhibited shorter delays vs. the peak MC signal than vs. the peak EMG signal. While the head angular acceleration counteracting the flexion delayed 15.0 ± 32.6 ms vs. the peak MC signal and 76.5 ± 45.5 ms vs. the peak EMG signal on average, the peak head rotation delayed more, 72.6 ± 53.0 ms vs. the MC signal and 134.1 ± 61.1 ms vs. the EMG signal, as also evident from Table 4. The neck flexion thus continued after the peak angular acceleration and peak neck muscle force, together with the lengthening of the neck extensors while electrical activity decreases. Comparison of the peak head rotation timing (Table 4) with the EMG time history (Figure 3i) shows that return motion of the head mostly happened with non-activated extensors, with small negative angular acceleration still present (Figure 3d).

For each sled test performed, based on data pairs recorded in 0–900 ms with time step 1 ms, the Pearson’s correlation coefficient $r_{P}$ was determined between the estimated neck muscle force and the MC sensor indentation force (Table 7). Fisher z′ transformation was used to determine the average correlation coefficients. The Kolmogorov-Smirnov test confirmed normal distribution of z′ for each severity level. The mean coefficients $r_{P}$ (*N* = 20) for the three severity levels were not significantly different. The overall mean correlation coefficient for all tests was ${\bar{r}}_{P}$ = 0.821 (*N* = 60), which implies strong linear correlation of the MC sensor indentation force to the neck muscle force.

For Subject 3, the correlation coefficients were considerably lower compared to the others, 0.381 < $r_{P}$ < 0.513. Detailed examination showed a pronounced startle response of Subject 3, with the peak MC indentation force preceding the peak neck muscle force by 59.6 ms on average (48–73 ms). Subject 3 also exhibited larger amplitudes of the dynamic response compared to the average across the subjects in each severity level (Table 6), the peak head rotation (39.9°, 48.3°, 55.7°) and timing (465.0 ms, 395.0 ms, 356.5 ms), the peak head excursion (20.4 cm, 20.5 cm, 22.9 cm) and timing (397.0 ms, 348.5 ms, 298.5 ms).

**Figure 4:**
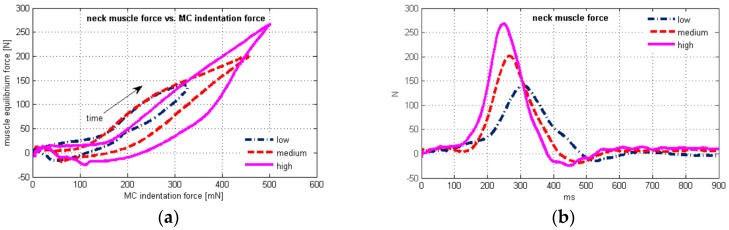
(**a**) Plot of the average muscle force vs. the average MC sensor indentation force; (**b**) time history of the estimated neck muscle force.

Plots of the average neck muscle force vs. time and vs. the average MC sensor indentation force are depicted in Figure 4 for the three test conditions. The scatter plot shows hysteretic behaviour (Figure 4a), where the upper and the lower branch of the hysteresis are sharply separated at the peak values, as expected due to the very small difference in peak timing of the neck muscle force and the MC signal (Table 6).

The peak EMG signal preceded the peak neck muscle force 61.8 ± 37.4 ms on average (Table 5), and was located on the upper branch of the hysteresis (Figure 4a), while electrical activity of extensor muscles was greatly reduced on the lower branch. At the end section of the hysteresis, negative values of the neck muscle force occurred (Figure 4b), together with the head return motion towards the neutral posture (Figure 3b).

## 4. Discussion

The results presented showed high linear correlation between the MC sensor signal and the neck muscle force during frontal impact. Previous research showed linear correlation between the muscle force of biceps brachii and the MC sensor indentation force in isometric conditions [39]. In our study, highly linear correlation was also found for neck loads during low-velocity frontal impact (Table 7). Considerably weaker linear correlation for an individual volunteer (Subject 3) may affect the overall correlation, since the Pearson’s coefficient is known to be sensitive to outlier data [47].

The results (Table 4) indicate stronger correlation of the MC signal for all peak values and timing of the *OC* loads, compared to the EMG signal. The correlation was evident in the cases of the shear force and the bending moment. The axial force timing did not correlate to EMG nor MC signals, while the later was weakly correlated to the axial force magnitude.

The neck load estimation was based on the inverse dynamics approach, where several factors could influence the results. We assumed that the muscle force of the neck extensors generates the moment around *OC*, maintaining dynamic equilibrium in neck flexion and extension. Similar to previous studies [6,36,48], there is very good agreement between time histories of the *OC* shear force and bending moment in low-velocity frontal impacts. The acceleration of the head *CG* causes the reaction force at *OC*, as well as the bending moment, due to the *CG* position with respect to *OC*. The distance between *CG* and *OC* is longer in the local z′-direction than in the x′-direction (Figure 2, Table 2), resulting in the shape of the bending moment and the shear force curves (Figure 3f,g). Peak axial loads were comparably lower in the impact conditions performed, without the order of magnitude excessing gravitational effects.

Previous studies [5,49] on frontal impacts identified muscle activation as a significantly more important parameter than osteoligamentous stiffness. The spine ligaments exhibit characteristic behaviour with small stiffness in the “toe” region and a nearly linear response in the elastic region [50], which results in low bending stiffness in the physiological range of motion of cervical segments, including skull-C2 [16,51,52]. Therefore, the bending moment is mostly counteracted by the neck extensor muscles, even more so in the low-severity frontal impacts performed in our study.

According to Vasavada et al. [53], the capability of a muscle to generate moment depends on intrinsic muscle characteristics influencing the maximum muscle force, moment arm (the line of muscle action with respect to the *OC* axis of rotation) and neural activation. Based on numerical simulations, they estimated the capability of isometric force and moment generation for individual neck muscles. Superficial neck muscles with the biggest moment arm in neck flexion, in the skull-C2 segment, are trapezius, splenius, semispinalis, with the moment arms decreasing in the flexed position. The suboccipital muscles can generate comparably low maximum force. Kumar et al. [42] estimated that the normalized peak EMG activity of trapezius can be up to 79% of the maximum voluntary contraction at low-severity impacts. That implies that trapezius has the most significant force generation ability. On the other hand, Ólafsdóttir et al. [37] estimated the role of trapezius as minor. Considering their study was based on volunteer testing at ~1.5 g, it implies that deeper neck muscles were active in stabilizing the head, and no major displacements and rotations occurred.

Several studies emphasized the importance of providing correct muscle path and moment arm in the analysis of the dynamic response in frontal impacts [54,55,56]. The moment arms of semispinalis and splenius capitis change at a very similar rate to trapezius, while for the rectus capitis posterior major the change is small [34]. It is reasonable to assume that the moment arm variations do not change significantly the load distribution among the neck extensors, but the cumulative neck extensor force increases as the moment arm shortens. Gao et al. [57] observed the same level of the peak load and timing for trapezius and semispinalis muscles, suggesting that measuring the stress of the trapezius muscle can also indicate the dynamic response for other neck extensors. With measuring the muscle tension on upper trapezius, muscle wrapping is already taken into account, providing a more favourable input parameter for estimating neck loads during frontal impacts.

The moderate correlation between the MC signal and peak head excursion, $r_{S}$ = 0.444 for peak magnitude and $r_{S}$ = 0.542 for timing (Table 4), is in accordance with the study by Hedenstierna et al. [26] that showed that the highest neck muscle strains occur at maximum head excursion, recognizing the need for a combination of the EMG signal and muscle strain measurement. Indeed, the head excursion delay vs. the peak MC signal was on average only 18.7 ± 36.7 ms (Table 5). The correlation of the MC signal was higher for the head acceleration timing, $r_{S}$ = 0.753, compared to $r_{S}$ = 0.594 for the EMG signal. Mean timing of peak magnitudes (Table 4) showed time lag of some head motion parameters vs. EMG and MC signals, which may effectively lower the Spearman’s correlation coefficients. However, there was no significant difference (*p* ≥ 0.4748) between the mean timing of the peak neck muscle force and MC indentation force at any severity level.

Since the axial force was rather low in magnitude, the neck bending moment was primarily affected by the angular acceleration and the shear force, where the average delay (*N* = 60) vs. the MC sensor was 15.0 ± 326 ms for the angular acceleration and −23.1 ± 24.0 ms for the shear force (Table 5). The average time delay of the peak bending moment was −3.4 ± 29.1 ms. An additional factor to influence the neck muscle equilibrium force was the moment arm dependent on the head rotation, resulting in the very small time lag between the neck muscle force and the MC signal, 0.2 ± 30.6 ms on average. That is substantially different from the average measured delay of the neck muscle force vs. the EMG signal, 61.8 ± 37.4 ms.

The time histories of the EMG signal (Figure 3i) and MC indentation force showed initial neck muscle activity, increased with impact severity, with the peak magnitude timing ~95–115 ms. However, the EMG onset of the neck muscles for an unaware occupant is no faster than 64 ms after the impact, according to Siegmund et al. [58]. Simultaneously, small neck extension occurred prior to the neck flexion, as seen from the head rotation plot (Figure 3b). This behaviour can most likely be attributed to the startle response of the volunteers anticipating the impact. The startle response can be triggered in pre-crash situations by visual, acoustic, vestibular, or somatosensory stimuli, and is characterised by short contraction of the upper body muscles and extension of the spine [15,59]. Similar to our observations, pre-impact activity of the neck muscles and head kinematics attributed to bracing was reported by Pelletiere et al. [60] and McGehee et al. [61].

The increased electrical activity of the neck muscles during early impact, up to ~160 ms, was accompanied by only a small increase of the MC signal. A possible reason is that the EMG electrodes also detected electrical activity of deeper neck muscles, which have a predominantly stabilizing function, but their activity cannot be measured directly by the MC sensor. That would confirm the observations of Ólafsdóttir et al. [37], who used needle electrodes for deep muscles. Another possible reason is that the neck extension from the neutral posture might decrease the surface tension at the MC sensor attachment location. It is also known that the muscle tension is not proportional to the EMG signal, due to its dependence on recruitment of the muscle units and firing rate in a particular muscle, where the phase cancellation occurs at an increased firing rate.

The initial neck muscle activity measured by the MC sensor prior to the neck flexion is reflected also in the flat section at the beginning of the hysteresis (Figure 4a). The equilibrium neck muscle force was estimated based on the inverse dynamics analysis of the head motion, yet neglected the muscle tension stabilizing the posture but not producing any motion. The estimated negative force in neck extensors at the end of the hysteresis could result from the flexion moment generated by the neck flexors, decelerating the head during the motion towards the neutral posture.

The steepness of the lower branch of the hysteresis further from the peak value indicates a higher rate of change of the neck muscle force with respect to the MC indentation force. The muscle tension remained comparably high, while the neck equilibrium force decreased. Partly, some residual muscle tension after the peak EMG signal and decrease (Figure 3i) may be attributed to the slower muscle deactivation, which is also evident from comparably high values of the MC sensor signal (Figure 3j). This observation agrees with Winters and Stark [62], who described the muscle deactivation process as slower than the activation. The importance of muscle deactivation in the dynamic response was also recognized by Brolin et al. [63].

Kallemeyn et al. [64] performed non-destructive experiments on C2–C7 osteoligamentous specimens and observed approximately 30° flexion and ~1 Nm moment, with mild hysteretic behavior. Considering the results of our study, the osteoligamentous stiffness of the spine and joint friction could only contribute to the observed hysteresis of the neck muscle force vs. muscle tension to a small degree.

Maximum head rotation occurred significantly after the peak EMG and peak MC signals (Table 4 and Table 6). Although the precision of the adopted model of the head-neck complex is somewhat compromised, compared to a highly biofidelic finite element model, it can be stated that the neck extensor muscles were subject to lengthening most of the head positive rotation phase (Figure 3b). This section of the upper branch of the hysteresis curve (Figure 4a) implies stronger linear correlation of the muscle force vs. the MC signal than for the whole sled test duration (Table 7). The increased steepness after the peak neck force (Figure 4a) occurred is accompanied with a decrease of the EMG signal (Figure 3i) and head rotation (Figure 3b). A possible reason to be considered is the muscle force-velocity relation [14], where muscle force changes at a higher rate when the lengthening rate decreases towards zero. The higher rate of the muscle force change can also result from the passive tension component, as the muscle force-length relation suggests. Additionally, the neck flexors act synergistically during the retraction, slowing down the head velocity to stabilize it in the neutral posture. The net *OC* bending moment estimated from the inverse dynamics is thus reduced, while the neck extensor force and the muscle tension measured by the MC sensor may be higher. Further research should consider a detailed model of the head-neck complex that would enable more precise analysis of the muscle geometry during the dynamic response, to quantify the effects of force-length and force-velocity relations in muscle modelling.

## 5. Conclusions

The role of the muscle load and activity in impact biomechanics is a challenging topic due to the troublesome experimental assessment and numerical modelling. Active muscle response of the vehicle occupant can affect the body dynamics and possible injuries, particularly in collision avoidance manoeuvres and low-severity collisions. Understanding the active and passive muscle-tendon complex behaviour that influences the human body motion is essential for analysis and design of vehicle restraint systems. Volunteer-based studies are inherently limited with the ability to measure muscle loads in vivo. Muscle forces can be estimated based on inverse or forward dynamic analysis of a human body model, compared to experimental data on dynamic response, where many input parameters assumptions are needed. Estimating the muscle mechanical action based on EMG may be subject to additional difficulties regarding the muscle electrical activity measuring and post-processing of the data. While muscle effort can be estimated under isometric or close to isometric loads, impact conditions introduce increased uncertainty.

In our study, we used the inverse dynamics analysis to estimate the loads on the neck musculature of the volunteers during a low-severity frontal impact. The estimated neck muscle force was compared to the data from the EMG and MC sensor for mechanomyography. The study showed strong linear correlation between the MC sensor indentation force and the neck muscle force in frontal impacts, with close to zero delay between the peak muscle force and MC sensor signal. The indentation force of the MC sensor represents an independent parameter from the EMG signal, being focused on the mechanical effect of a muscle rather than the muscle electrical activity. Also, the passive component of the muscle-tendon complex force cannot be estimated by the EMG itself. The MC sensor can efficiently measure activity of the superficial neck muscles that contribute the main action to the impact response. The deeper muscles with a stabilizing role can still be accessed with EMG needle electrodes only.

The mechanomyography offers the possibility to complement and overcome some difficulties of EMG, commonly used in volunteer testing of human body dynamics and interaction with a vehicle. The potential usability of MC sensors includes analysis of the human body response in low-severity impact conditions, development of restraints and active safety systems, as well as ergonomy of vehicle interiors.

## Figures and Tables

**Table 1:** Sled deceleration pulse data for low, medium, and high impact severity (mean ± SD).

	Low	Medium	High
impact velocity (km/h)	7.6 ± 0.39	9.5 ± 0.50	11.5 ± 0.38
max. deceleration (g)	1.7 ± 0.05	2.6 ± 0.07	3.8 ± 0.11

**Table 2:** Geometrical and mass properties of the head.

Test Subject	Center of Gravity Location (cm)	Occipital Condyle Location (cm)	Mass m (kg)	Moment of Inertia $I_{y\prime }^{CG}\;\left(\mathrm{kg}\cdot cm^{2}\right)$
Males: 1, 2, 4, 5, 7, 9, 10	$x^{\prime CG}=0.83$ $z^{\prime CG}=-3.12$	$x^{\prime OC}=-0.889$ $z^{\prime OC}=3.175$	4.323	226.05
Females: 3, 6, 8	$x^{\prime CG}=0.47$ $z^{\prime CG}=-2.92$	$x^{\prime OC}=-0.889$ $z^{\prime OC}=3.175$	4.125	198.5

**Table 3:** Head horizontal position and orientation at low, medium, and high impact severity (mean ± SD).

	Low	Medium	High
head position *x* (cm)	1.9 ± 2.5	0.8 ± 2.0	0.2 ± 2.4
Frankfurt plane (°)	2.2 ± 5.3	−2.5 ± 5.7	−3.0 ± 6.9

**Table 4:** Characteristic parameters of head motion, EMG and MC sensor signals, mean ± SD; Spearman's correlation coefficients $r_{S}$ (*N* = 60) with corresponding *p*-values ^1^ between the head motion parameters and EMG, MC sensor data for low (1.7 g), medium, and high impact severity.

	Low	Medium	High	$r_{S}$ (EMG)	*p*-Value $r_{S}$ (EMG)	$r_{S}$ (MC)	*p*-Value $r_{S}$ (MC)
Peak values							
head excursion (mm)	141.1 ± 39.9	168.1 ± 34.9	178.5 ± 26.5	**0.285**	**0.0271**	**0.444**	**0.0003**
head rotation (°)	17.8 ± 9.9	26.6 ± 12.5	31.7 ± 12.3	0.153	0.2423	**0.397**	**0.0016**
head acceleration (m/s^2^)	32.7 ± 2.9	41.7 ± 3.8	50.7 ± 4.7	**0.715**	**<0.0001**	**0.500**	**<0.0001**
angular acceleration (rad/s^2^)	−52.1 ± 23.9	−97.0 ± 30.0	−122.6 ± 36.7	−0.504	**<0.0001**	**−0.553**	**<0.0001**
axial force (N)	14.4 ± 12.0	7.5 ± 16.7	3.3 ± 16.3	−0.057	0.6643	**−0.363**	**0.0043**
shear force (N)	103.9 ± 12.8	139.7 ± 19.3	174.7 ± 26.4	**0.670**	**<0.0001**	**0.510**	**<0.0001**
bending moment (Nm)	−6.28 ± 1.03	−8.98 ± 2.09	−11.42 ± 2.56	**−0.567**	**<0.0001**	**−0.515**	**<0.0001**
neck muscle force (N)	146.1 ± 28.4	213.6 ± 61.4	277.9 ± 77.9	**0.513**	**<0.0001**	**0.575**	**<0.0001**
EMG (µV)	63.0 ± 13.2	86.6 ± 17.3	108.2 ± 23.2				
MC sensor (mN)	381.2 ± 181.3	481.5 ± 174.8	572.4 ± 214.3				
Timing of peak values							
head excursion (ms)	332.0 ± 27.7	289.5 ± 26.1	264.8 ± 17.9	**0.411**	**0.0011**	**0.542**	**<0.0001**
head rotation (ms)	387.5 ± 41.5	341.9 ± 41.3	318.4 ± 45.1	0.154	0.2386	**0.329**	**0.0100**
head acceleration (ms)	289.1 ± 16.2	239.6 ± 13.4	220.9 ± 10.6	**0.594**	**<0.0001**	**0.753**	**<0.0001**
angular acceleration (ms)	328.2 ± 16.3	290.4 ± 28.7	264.7 ± 22.2	0.349	**0.0062**	0.563	**<0.0001**
axial force (ms)	327.9 ± 85.0	309.9 ± 96.9	290.9 ± 85.9	0.047	0.7222	0.158	0.2258
shear force (ms)	293.1 ± 18.1	243.9 ± 11.9	223.6 ± 9.4	**0.591**	**<0.0001**	**0.742**	**<0.0001**
bending moment (ms)	308.5 ± 17.9	264.9 ± 17.4	246.1 ± 11.3	**0.557**	**<0.0001**	**0.638**	**<0.0001**
neck muscle force (ms)	311.4 ± 17.3	268.5 ± 19.8	250.8 ± 14.4	**0.513**	**<0.0001**	**0.600**	**<0.0001**
EMG (ms)	244.0 ± 42.4	205.3 ± 34.9	196.0 ± 28.9				
MC sensor (ms)	315.9 ± 25.4	267.3 ± 22.1	246.6 ± 21.0				

^1^ Bold values indicate statistical significance (*p* < 0.05).

**Table 5:** Time delay between peak values of characteristic parameters of head motion vs. the EMG and MC sensor signals for low (1.7 g), medium (2.6 g) and high (3.8 g) impact severity (mean ± SD).

Delay (ms)	Low		Medium		High	
	EMG	MC	EMG	MC	EMG	MC
head excursion	87.9 ± 41.9	16.0 ± 41.8	84.2 ± 46.2	22.1 ± 37.9	68.7 ± 36.8	18.0 ± 31.3
head rotation	143.5 ± 54.1	71.6 ± 56.0	136.6 ± 86.0	74.5 ± 51.5	122.4 ± 61.7	71.7 ± 54.1
head acceleration	45.0 ± 38.5	−26.8 ± 25.1	34.2 ± 33.9	−27.8 ± 21.0	24.8 ± 29.1	−25.8 ± 23.6
angular acceleration	84.2 ± 50.5	12.3 ± 35.5	80.3 ± 48.9	18.2 ± 34.1	65.0 ± 35.6	14.4 ± 29.5
axial force	83.9 ± 105.2	12.0 ± 98.7	104.6 ± 105.7	42.5 ± 98.5	94.8 ± 92.7	44.1 ± 87.5
shear force	49.0 ± 39.4	−22.8 ± 26.8	38.6 ± 33.3	−23.4 ± 22.0	27.6 ± 28.8	−23.0 ± 24.1
bending moment	64.5 ± 38.1	−7.3 ± 31.9	59.6 ± 37.7	−2.4 ± 29.5	50.0 ± 29.5	−0.5 ± 26.8
neck muscle force	67.3 ± 37.9	−4.5 ± 31.7	63.2 ± 40.9	1.2 ± 30.8	54.7 ± 33.9	4.1 ± 30.1

**Table 6:** Results of paired *t*-test of peak magnitude timing of head motion parameters and *OC* loads vs. timing of the peak MC and EMG signals. The samples for low (1.7 g), medium (2.6 g), and high (3.8 g) impact severity were tested for *p*-values ^1^.

	Low		Medium		High	
	vs. EMG	vs. MC	vs. EMG	vs. MC	vs. EMG	vs. MC
head excursion	**<0.0001**	0.0635	**<0.0001**	**0.0064**	**<0.0001**	**0.0058**
head rotation	**<0.0001**	**<0.0001**	**<0.0001**	**<0.0001**	**<0.0001**	**<0.0001**
head acceleration	**<0.0001**	**0.0001**	**0.0002**	**<0.0001**	**0.0012**	**0.0001**
angular acceleration	**<0.0001**	0.1373	**<0.0001**	**0.0272**	**<0.0001**	**0.0416**
axial force	**0.0020**	0.5921	**0.0003**	0.0684	**0.0002**	**0.0360**
shear force	**<0.0001**	**0.0012**	**<0.0001**	**0.0001**	**0.0004**	**0.0004**
bending moment	**<0.0001**	0.3142	**<0.0001**	0.7203	**<0.0001**	0.9245
neck muscle force	**<0.0001**	0.5316	**<0.0001**	0.8637	**<0.0001**	0.5475

^1^ Bold values indicate statistical significance (*p* < 0.05).

**Table 7:** Pearson’s correlation coefficients $r_{P}$ between the neck muscle equilibrium force and the MC sensor indentation force for low (1.7 g), medium (2.6 g) and high (3.8 g) severity levels. Mean correlation for all the tests performed is ${\bar{r}}_{P}$ = 0.821 (*N* = 60).

Subject	Low		Medium		High	
	Test 1	Test 2	Test 1	Test 1	Test 2	Test 1
1	0.776	0.936	0.824	0.886	0.851	0.849
2	0.631	0.739	0.724	0.844	0.877	0.722
3	0.381	0.400	0.512	0.500	0.493	0.513
4	0.829	0.947	0.827	0.950	0.877	0.842
5	0.773	0.746	0.910	0.821	0.899	0.913
6	0.883	0.881	0.801	0.807	0.825	0.855
7	0.768	0.857	0.738	0.762	0.691	0.809
8	0.780	0.781	0.766	0.822	0.710	0.783
9	0.899	0.886	0.838	0.883	0.839	0.838
10	0.944	0.920	0.942	0.721	0.856	0.862
* mean z′		1.181		1.157		1.143
mean $r_{P}$		0.827		0.820		0.815

* Fisher z′ transformation.
